# Enhancing Cadmium Tolerance and Pea Plant Health through *Enterobacter* sp. MN17 Inoculation Together with Biochar and Gravel Sand

**DOI:** 10.3390/plants9040530

**Published:** 2020-04-20

**Authors:** Muhammad Naveed, Adnan Mustafa, Samar Majeed, Zainab Naseem, Qudsia Saeed, Abdulhameed Khan, Ahmad Nawaz, Khurram Shehzad Baig, Jen-Tsung Chen

**Affiliations:** 1 Institute of Soil and Environmental Sciences, University of Agriculture Faisalabad, Faisalabad 38000, Pakistan; adnanmustafa780@gmail.com (A.M.); samarmajeed@gmail.com (S.M.); zainabnaseem747@gmail.com (Z.N.); 2 National Engineering Laboratory for Improving Quality of Arable Land, Institute of Agricultural Resources and Regional Planning, Chinese Academy of Agricultural Sciences, Beijing 100081, China; 3 College of Natural Resources and Environment, Northwest Agriculture and Forestry University, Yangling 712100, China; qudsia.saeed@nwafu.edu.cn; 4 Department of Biotechnology, University of Azad Jammu and Kashmir, Muzaffarabad 13100, Azad Jammu and Kashmir, Pakistan; abdulhameed.khattak81@gmail.com; 5 Integrated Pest Management Laboratory, Department of Entomology, University of Agriculture, Faisalabad 38000, Pakistan; nawazrajpoot65@gmail.com; 6 Soil Fertility Institute, Lahore 53700, Punjab, Pakistan; ksb2004@gmail.com; 7 Department of Life Sciences, National University of Kaohsiung, Kaohsiung 811, Taiwan

**Keywords:** immobilizing agents, *Pisum sativum*, cadmium toxicity, endophytic bacteria, contaminated soil, plant health

## Abstract

Contamination of soils with heavy metals, particularly cadmium (Cd), is an increasingly alarming environmental issue around the world. Application of organic and inorganic immobilizing amendments such as biochar and gravel sand in combination with metal-tolerant microbes has the potential to minimize the bioavailability of Cd to plants. The present study was designed to identify the possible additive effects of the application of *Enterobacter* sp. MN17 as well as biochar and gravel sand on the reduction of Cd stress in plants and improvement of growth and nutritional quality of pea (*Pisum sativum*) plants through the reduction of Cd uptake. Pea seeds were surface sterilized then non-inoculated seeds and seeds inoculated with *Enterobacter* sp. MN17 were planted in artificially Cd-polluted soil, amended with the immobilizing agents biochar and gravel sand. Application of biochar and gravel sand alone and in combination not only improved the growth and nutritional quality of pea plants by in situ immobilization but also reduced the uptake of Cd by plant roots and its transport to shoots. However, microbial inoculation further enhanced the overall plant health as well as alleviated the toxic effects of Cd on the pea plants. These soil treatments also improved rates of photosynthesis and transpiration. The combined use of biochar and gravel sand with bacterial inoculation resulted in an increase in plant height (47%), shoot dry weight (42%), root dry weight (57%), and 100 seeds weight (49%) as compared to control plants in Cd contaminated soil. Likewise, biochemical constituents of pea seeds (protein, fat, fiber, and ash) were significantly increased up to 41%, 74%, 32%, and 72%, respectively, with the combined use of these immobilizing agents and bacterium. Overall, this study demonstrated that the combined application of biochar and gravel sand, particularly in combination with *Enterobacter* sp. MN17, could be an efficient strategy for the remediation of Cd contaminated soil. It could support better growth and nutritional quality of pea plants.

## 1. Introduction

Soil contamination is a global problem with risks associated with human health and the environment. Heavy metals such as lead (Pb), cadmium (Cd), zinc (Zn), mercury (Hg), arsenic (As), silver (Ag), chromium (Cr), copper (Cu), and iron (Fe) are the most challenging, emergent soil contaminants being toxic to plants and dangerous to humans when consumed in agricultural produce [1,2,3,4]. Anthropogenic activities such as manufacturing industries, mining, and uncontrolled use of materials containing heavy metals in agriculture (including but not limited to fertilizers, pesticides, industrial effluents, and sewage sludge) are the most common sources of heavy metals in soil [5]. Control of these elements is considered a target for achieving sustainable environmental goals.

Cadmium, one of the target metals, is a nondegradable, toxic, and nonessential element for plants. It enters the environment naturally through volcanic eruptions, rock weathering, and forest fires and anthropogenically through various activities such as ceramic waste materials, ore mining, processing of Ni–Cd batteries, plastic manufacturing, pigments, cement, and electroplating operations [6]. However, the most common human activities causing contamination of soil with Cd are the use of sewage sludges, fertilizers, atmospheric depositions, and metallurgical industries. By virtue of its highly mobile nature in soil, it readily accumulates in plant roots and other edible plant parts [7] causing metabolic, physiological, and biochemical disorders through reduced production of chlorophyll, uptake of nutrients, and photosynthesis, which results in stunted plant growth and greatly reduced crop production [8,9,10,11,12,13,14,15]. Moreover, long-term exposure and intake of food contaminated with Cd is a severe threat to human health [16,17]. Therefore, it is imperative to remediate Cd-polluted soils to restore the ecological functioning of such soils. 

Various approaches ranging from physical excavation to chemical extraction and biological remediation had been employed, but all these approaches have shortcomings in terms of high cost, negative environmental impacts, and loss of major nutrients required for normal plant growth [18,19,20,21,22]. On the other hand, in situ immobilization measures have been found to be a promising means of remediating Cd-polluted soils and of decreasing its bioavailability to plants, to be low cost, and to be environmentally appropriate [23]. A number of metal immobilizing agents including biochar, inorganic zeolite, metal oxides and phosphates, and composts have been studied to ameliorate Cd phytotoxicity in soils contaminated with heavy metals [13,14,15,24,25,26,27].

Biochar produced under air-limited thermal degradation of C-rich biomass [28] has been proven to effectively remove toxic metals from polluted environments, through adsorption and metal-stabilization mechanisms, a result of its large surface area, ion complexation, exchange, and precipitation properties [12,29,30,31,32]. However, success is highly determined by soil type, biochar surface properties, feedstock, pyrolytic temperature, and rate of application [33,34]. Combining biochar with other treatments such as metal-tolerant microbes has resulted in increased efficacy, cost-effectiveness, and environmental conservation. 

Plant growth-promoting bacteria are frequently used to improve crop production and are applicable to diverse agricultural settings [35]. The rhizosphere as well as endophytic bacteria can be used, and it is possible that the latter may be the more effective in promoting plant growth due to their intimate relationship with plant tissues [36]. Apart from growth promotion, these bacteria may improve the resistance of plants against pathogens, drought, and heavy metals stresses [37]. Such bacteria colonize plant roots and enhance their growth by multiple mechanisms. These microbes, when applied to contaminated soils, ameliorate stress by reducing heavy metal uptake via decreased availability in the rhizosphere [38,39]. The low availability of these metals in rhizospheric soils, in turn, favors root elongation and enhances plant growth [15]. The metal-resistant microbes may immobilize heavy metals through the production of exopolysaccharides, siderophores, and metal phosphates or through enhanced rhizosphere acidification [40,41].

In Pakistan, vegetables are mainly grown in areas irrigated with sewage water contaminated with heavy metals [42,43]. Consequently, there is an urgent need to explore techniques for minimizing the bioavailability of heavy metals to plants, especially vegetables. There is limited work on the impact of biochar and gravel sand in combination with metal-tolerant microbes to mitigate the adverse effects of heavy metals on plants. In addition, the effect of the combined use of endophytic bacteria and organic amendments such as biochar on the growth, physiochemical attributes, and nutrient concentrations in plants needs further study. Therefore, the present research was designed to investigate the potential roles of biochar and gravel sand with and without the inoculation of the endophytic plant growth-promoting bacterium *Enterobacter* sp. MN17 on the growth, gaseous exchange attributes, biochemical constituents, and nutrient content of pea plants grown in Cd-spiked sandy clay loam soil.

## 2. Results 

### 2.1. Plant Biomass Production 

**Figure 1:**
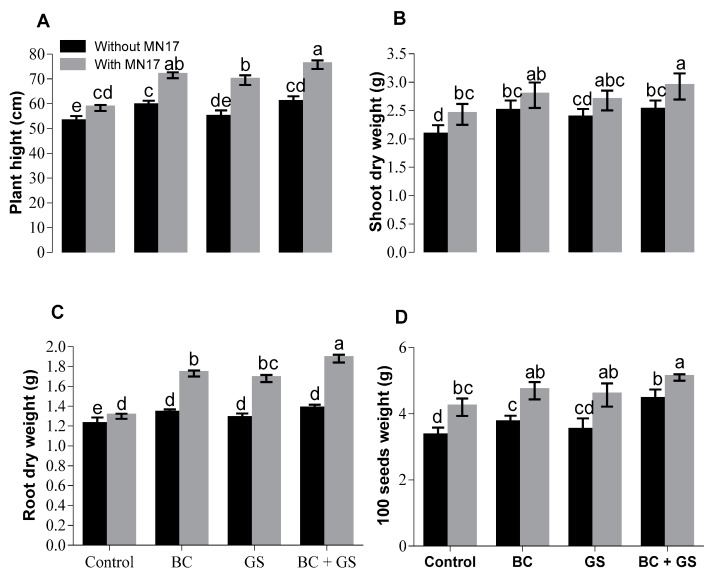
Effects of immobilizing agents (biochar and gravel sand) with and without seed inoculation of *Enterobacter* sp. MN17 on growth parameters of pea plant under Cd-contaminated soil conditions. Bars sharing the same letters in each graph do not differ significantly at *p* < 0.05. The values are mean ± S.D. (*n* = 3). BC: biochar; GS: gravel sand; BC + GS: biochar + gravel sand; MN17: *Enterobacter* sp. MN17.

The application of biochar and gravel sand with and without bacterial inoculation considerably improved plant height as compared to the control in soil containing Cd (Figure 1A). The effect of these immobilizing agents was more pronounced when combined with bacterial inoculation as compared to their single application. The increase in growth (plant height) compared with the control following the application of biochar was 16%, of gravel sand was 5%, and of biochar as well as gravel sand was 16%. Inoculation with *Enterobacter* sp. MN17 alone showed an 11% increase in plant height as compared to the control, while when inoculation was combined with biochar, the increase was 40%; with gravel sand, was 32%; and with both, was 47%. Shoot and root dry weights were increased when biochar, gravel sand, and their combination were used without inoculation (Figure 1B,C). Shoot and root dry weights were further enhanced when biochar, gravel sand, and their combination were used together with inoculation. The effect was greatest for root dry weight; inoculated plants with biochar and gravel sand showed 57% higher root dry weight than the controls with no treatments (Figure 1B,C). The use of biochar, gravel sand, and combination without bacterial inoculation also resulted in an increase in 100-seed weight as compared to the control, with a further increase (49%) observed when plants with these additives were inoculated (Figure 1D).

### 2.2. Physiological Gaseous Exchange Attributes 

**Figure 2:**
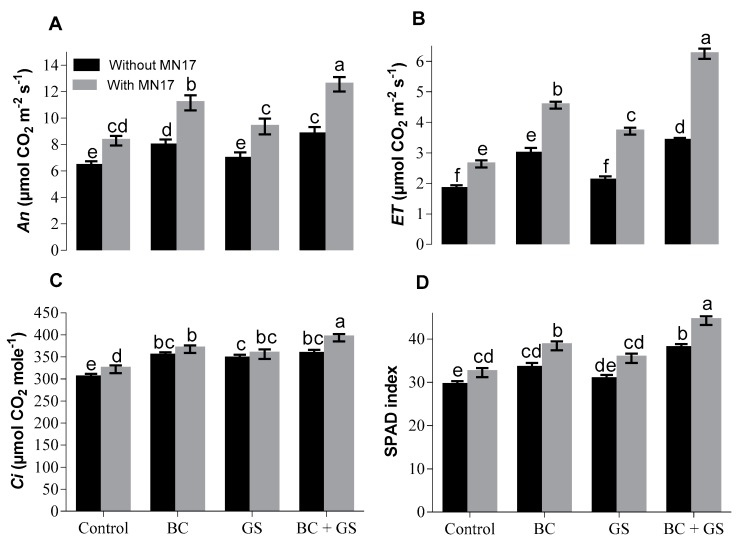
Effects of immobilizing agents (biochar and gravel sand) with and without seed inoculation of *Enterobacter* sp. MN17 on physiological parameters of pea plants under Cd-contaminated soil conditions. Bars sharing the same letters in each graph do not differ significantly at *p* < 0.05. The values are mean ± S.D. (*n* = 3). BC: biochar; GS: gravel sand; BC + GS: biochar + gravel sand; MN17: *Enterobacter* sp. MN17; An; net assimilation rate, ET; evapotranspiration rate, Ci; internal carbon dioxide concentration, and Soil Plant Analysis Development (SPAD) index.

Application of biochar but not gravel sand improved assimilation rate, while the combined use of biochar, gravel sand, and inoculation resulted in the greatest increase of assimilation rate (98%) (Figure 2A). The responses to the various treatments were similar for evapotranspiration rates with, again, the combined use of biochar, gravel sand, and inoculation giving the highest increase (87%) as compared to the control (Figure 2B). In the case of internal CO_2_ concentration, individual treatments without inoculation showed an increase while those treatments with the addition of inoculation showed a further small increase (Figure 2C). Data for the soil and plant analysis development (SPAD) index also showed that increases in the index were greater when the biochar and gravel sand treatments were accompanied by bacterial inoculation (Figure 2D).

### 2.3. Biochemical Analysis

The protein content, fat, fiber, and ash of seeds were markedly affected by Cd treatment. However, the application of biochar and gravel sand alone and in combination significantly reduced the Cd-induced disturbances in these parameters specifically when combined with bacterial inoculation (Table 1). A 41% increase in protein over the control resulted from the combined use of biochar, gravel sand, and bacterial inoculation. These combined treatments also gave a 74% increase in fat, a 32% increase in fiber, and a 72% increase in ash content (Table 1).

### 2.4. Contents of Fe and Zn in Pea Seeds

The application of biochar and gravel sand with and without bacterial application also significantly increased the seed content of Fe and Zn from the plants grown in Cd-spiked soil (Table 1), while the combined application of biochar, gravel sand, and bacterial inoculation resulted in an 84% increase in Fe content and a 47% increase in Zn content of the seeds (Table 1). The bacterial inoculation alone showed an increase of 26% and 12% in Fe and Zn of peas seeds as compared to control.

### 2.5. Cd Concentration in Soil and Plant Tissues

**Figure 3:**
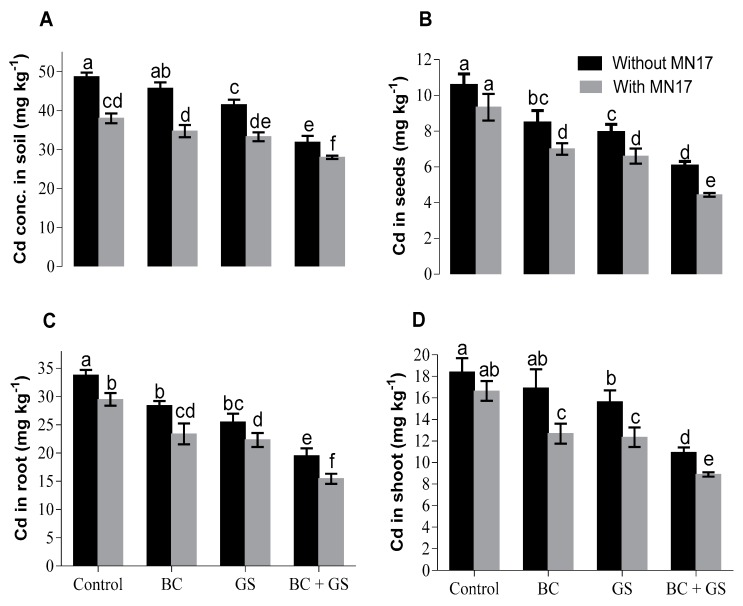
Effects of immobilizing agents (biochar and gravel sand) with and without seed inoculation of MN17 on Cd concentration in soil, seeds, root, and shoot tissues of peas grown in Cd-contaminated soil. Bars sharing the same letters in each graph do not differ significantly at *p* < 0.05. The values are mean ± S.D. (n = 3). BC: biochar; GS: gravel sand; BC + GS: biochar + gravel sand; MN17: *Enterobacter* sp. MN17

Initially, 60 mg Cd was added per kg of soil. Extractable Cd concentration in soil was reduced with the application of gravel sand but not with biochar applied alone. However bacterial inoculation alone resulted in a significant decrease in soil Cd. Biochar, gravel sand, and bacteria in combination resulted in the maximum decrease of 42% (28 mg Cd kg^−1^ soil). Bacterial inoculation alone showed a 22% decrease in soil Cd concentration over control (Figure 3A). The sole application of biochar or gravel sand resulted in a significant decrease in Cd content of seeds and roots, and gravel sand but not biochar had this effect also in shoots (Figure 3B–D). The greatest decreases (58%, 54%, and 52%) in plant tissues (seeds, root, and shoot) contents of Cd were seen when the biochar and gravel sand were combined with bacterial inoculation of seeds as compared to control.

### 2.6. Persistence of Inoculant Strain MN17 in the Rhizosphere, Pea Roots, and Shoots

**Figure 4:**
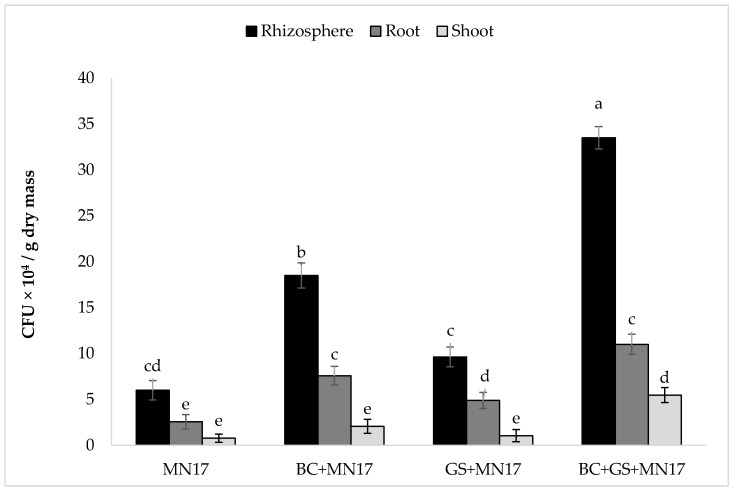
Persistence of the endophytic bacterial strain MN17 in the rhizosphere and presence in roots and shoots extracts of *P. sativum* grown in Cd-contaminated soil. Bars sharing the same letters do not differ significantly at *p* < 0.05. The values are mean ± S.D. (*n* = 3). BC: biochar; GS: gravel sand; BC + GS: biochar + gravel sand; MN17: *Enterobacter* sp. MN17.

The inoculant strain MN17 efficiently colonized the rhizosphere and was endophytic in the roots and shoots of pea plants grown in the Cd-contaminated soil (Figure 4). Bacterial colonization of the rhizosphere was increased by the application of biochar or by biochar and gravel sand but not by gravel sand alone (Figure 4). Similarly, the endophyte populations estimated from root extracts was improved in all three treatments but highest when biochar was present. Bacterial populations in shoots were not improved unless both biochar and gravel sand were applied. Inoculant strain CFU g^−1^ dry weight was recovered from the rhizosphere (3.35 × 10^5^), root interior (1.10 × 10^5^), and shoot interior (5.47 × 10^4^) where a combination of biochar, gravel sand, and MN17 was applied.

### 2.7. Pearson Correlation and Principal Component Analysis (PCA)

**Figure 5:**
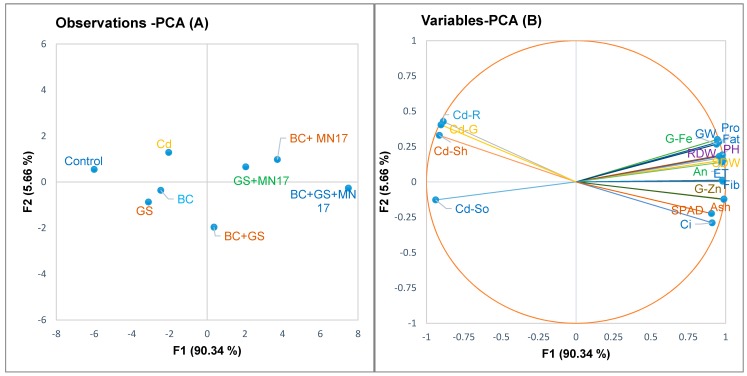
Principal component analysis of observations (left) and variables (right), whereby the first two components revealed 96% of the variability between the applied treatments and studied parameters of pea plant under Cd-stressed soil conditions. Observations are control; BC: biochar; GS, gravel sand; BC + GS, biochar + gravel sand; BC + GS + MN17, biochar + gravel sand inoculated with MN17; PH, plant height; SDW, shoot dry weight; RDW, root dry weight; GW, grains weight; Pro, protein; Fat, fat; Fib, fiber; Ash, ash; G-Fe, Fe concentration in grains; G-Zn, Zn concentration in grains; Cd-So, Cd concentration in soil; Cd-Sh, Cd concentration in shoots; Cd-R, Cd concentration in roots; Cd-G, Cd concentration in grains; Ci, internal carbon dioxide concentration; An, net assimilation rate: ET, evapotranspiration rate, and SPAD, chlorophyll (SPAD) contents.

Significant positive and negative correlations were observed among plant growth (plant height, shoot dry weight, root dry weight, and grains weight) and physiological (internal carbon dioxide concentration, net assimilation rate, evapotranspiration rate, and SPAD index) and biochemical parameters (protein, fat, fiber, and ash) along with Fe and Zn contents of pea grains and Cd concentrations in soil and plant tissues under Cd-contaminated soil conditions (Table 2). The score and loading plots of principal component analysis (PCA) are presented in Figure 5. Within the dataset, the first two components of PCA revealed maximum (96%) variation among all the studied parameters, of which PC1 explained 90.34% variation whereas PC2 explained 5.66% variation. Moreover, all of the applied treatments were successfully displaced with the first two components. This displacement of treatments provided a clear indication that the application of biochar and gravel sand alone and in combination had a significant ameliorative effect on all the studied attributes of pea plants relative to the control (Figure 5A). Here, PC1 was positively influenced by variables PCA (Figure 5B) having parameters pH, SDW, RDW, GW, Pro, Fat, Fib, Ash, G-Fe, G-Zn, Ci, An, ET, and SPAD), whereas PC2 was positively influenced by observations PCA containing (Cd-Soil, Cd-S, Cd-R, and Cd-G). Furthermore, a significantly negative correlation was found between the parameters of PC1 and PC2.

## 3. Discussion

This research demonstrated how the application of biochar and gravel sand, particularly in combination with inoculation with an endophytic *Enterobacter* sp. MN17, provides a cost-effective, environmentally friendly way of increasing yield and of reducing the Cd content of pea plants growing in Cd-contaminated soil. These results support published studies on other species showing that organic and inorganic soil amendments and microbial inoculation can improve plant growth on heavy metal contaminated soil [12,15,33,44,45,46]. Amongst the properties of biochar and gravel sand are that they adsorb Cd onto their surfaces and make it less available to plant roots [47,48]. The *Enterobacter* sp. might also have decreased plant-available Cd by reducing rhizospheric pH through the production of metal phosphates [15,41], through metal-chelating proteins, and/or by intracellular uptake of Cd [49,50]. 

The physiology of the pea plants was shown to be adversely affected by the concentration of Cd in the soil, and this resulted in plant growth parameters (plant height, root dry weight, shoot dry weight, and seed weight) showing a negative correlation with the available Cd in the soil. Application of biochar, gravel sand, or both and these treatments with the addition of bacterial inoculation resulted in a reduction of Cd-induced phytotoxic disturbances and improved growth. The reduction in the growth of crops might be because one of the toxic impacts of Cd is that it stunts plant roots, particularly the formation of the lateral roots, and this ultimately affects nutrient uptake and plant growth [14,15,51,52,53,54]. Further, Cd can occupy exchange sites and thus interfere with the plant nutrient uptake [55,56], or it can affect the soil microbial activity necessary for nutrient cycling [57,58]. In the present study, while improved plant growth parameters were observed with the application of biochar, gravel sand, or both, the maximum increase in growth was recorded with microbial inoculation along with these immobilizing agents. This might be in part due to the ability of *Enterobacter* sp. MN17 to grow on high Cd levels [15], but this endophytic bacterial strain has been shown to have several other beneficial plant-growth-promoting characteristics: phosphate solubilization, siderophore production, 1-aminocyclopropane-1-carboxylate-deaminase activity, indole-3-acetic acid, and exopolysaccharides production under both normal and stress conditions [36,59]. These properties may explain the improvement of plant growth in pots with inoculated seeds but no soil amendments (Figure 1).

An excess of heavy metals including Cd in soil or the plant body causes significant reductions in gaseous exchange parameters of plants [15,26,60,61,62,63,64]. We observed improvement in gaseous exchange parameters such as internal CO_2_ concentration, net assimilation rate, evapotranspiration rate, and SPAD index of pea plants growing in Cd-contaminated soil when soil additives and bacterial inoculation reduced the rhizosphere and plant concentrations of Cd (Figure 2 and Figure 3). The reduction in gaseous exchange parameters caused by Cd might be due to impaired stomatal physiology [65], resulting in lowered internal CO_2_ concentrations, net assimilation of photosynthates, and transpiration. Replacement of central Mg^2+^ of the chlorophyll molecule by Cd may also be deleterious and decreases the net assimilation rate.

The negative effects of Cd concentration on nutritional quality (Table 2) may be because of the interruption of absorption of essential plant nutrients through reduced root proliferation [66,67]. Bacterial inoculation may enhance nutrient uptake (Fe and Zn) through the production of various metabolites, e.g., organic acids which release fixed nutrients so they are available for plant utilization [68,69]. The *Enterobacter* sp. MN17 has the potential to chelate Fe through siderophore production and to solubilize Zn, thus enhancing the uptake of both nutrients, as shown in the present study. Cadmium uptake and allocation are also associated with divalent metal cations (including Fe and Zn) transporters; the higher uptake of these nutrients might be a result of direct competition with Cd. Further, biochar is also a source of macro- and micronutrients and can play a role in nutrients uptake through different mechanisms. Applied biochar, gravel sand, and bacterial strain MN17 might have evoked plant physiological adaptation to a stressed environment which subsequently enhanced the nutritional quality of the pea seeds [70].

Improved nutrient uptake results in higher nutritional qualities [71,72], and this was the case in our experiments as higher uptake of Zn and Fe, following application of biochar, gravel sand, and bacterial inoculation, provided for improved values of pea seeds quality parameters (protein, fat, fiber, and ash) in the Cd-spiked soil.

In the control treatment, a higher concentration of Cd in plants was observed with Cd spiking in the soil (Figure 3). The application of biochar and gravel sand alone and in combination resulted in decreased Cd uptake by pea plant roots and shoots. However, their combined application along with bacterial strain MN17 resulted in a maximum decrease in Cd uptake by pea plants. These results suggested a mutually bipartite relationship between the applied amendments and endophytic bacterium MN17. The decreased uptake of Cd in microbial inoculated plants under immobilizing agents might be due to the enhanced Cd immobilization in soil [13,14,15,47,73]. These results are substantiated by the findings of other researchers as well [74,75,76]. *Enterobacter* sp. MN17 probably had reduced the available fractions of Cd in soil by the formation of metal-chelating proteins and/or by intracellular uptake of Cd [15,49,50]. Lower availability of Cd may also be due to the aging effect of the applied treatments, which results in a decline in exchangeable/soluble fractions of metals over time caused by surface adsorption, precipitation, or complex formation.

World population growth means that, increasingly, crops will need to be grown on problematic soils. Cost-effective solutions are needed to avoid detrimental effects on plant growth and human health. Our results on the use of soil amendments and bacterial inoculation of peas add to the body of evidence that such treatments ameliorate the impact of excess soil Cd.

## 4. Materials and Methods

### 4.1. Soil Preparation, Seed Source, and Inoculation with Enterobacter sp. MN17

Soil samples were collected from the local Research Farm of Institute of Soil and Environmental Sciences (ISES), University of Agriculture, Faisalabad (UAF), Pakistan. It was air-dried and then ground to pass through a 2.0-mm sieve. Soil samples were analyzed for the physicochemical characteristics listed in Table 3. Soil particle size was analyzed following the method of Gee and Bauder [77]. The textural class analysis revealed that the texture was sandy clay loam. Saturation percentage, soluble carbonates (CO_3_^2−^), and bicarbonates (HCO_3−_) were measured according to the method given by Richards [78]. Organic matter (%) and total nitrogen in the soil were measured according to Moodie et al. [79], available phosphorous was measured using the Watanabe and Olsen [80] method, and available potassium was measured using the method of Simard [81] by Flame photometer (Jenway PFP7, UK). For Diethylenetriamine pentaacetic acid (DTPA) extractable Cd, 0.05 mol L^−1^ DTPA at pH 7.3 was used to measure Cd directly by atomic absorption spectrophotometery (AAS) (Perkin Elmer Aanalyst-100, USA).

A culture of the endophytic bacterial strain was taken from Environ. Sci. Lab., Inst. Soil Environ. Sci., University of Agriculture, Faisalabad. This strain was previously isolated from maize root tissues and is known to tolerate heavy metals and to improve the growth and yield of various crops under normal and abiotic stress conditions [15,36,59,82,83]. The culture of *Enterobacter* sp. MN17 used for seed inocululation was prepared by placing a loopful of inoculum into 100-mL tryptic soy broth in a 200-mL Erlenmeyer flask. This was incubated on an orbital shaker (VWR International GmbH, Austria) at 120 rpm for 24 hours at 28 + 2 °C. Harvesting of the culture and population establishment were performed as described by Saeed et al. [15]. Healthy seeds of pea plants were surface sterilized by following the method of Naveed et al. [82]. Seeds were then inoculated with the MN17 broth, peat, and clay mixed with a 10% sugar solution. Seeds were shaken until a fine layer of inoculum appeared on the seeds and were then dried in the laboratory overnight. Seeds of pea cultivar “Meteor Faisalabad” were very kindly provided by Vegetable Research Institute, Ayub Agricultural Research Institute, Faisalabad, Pakistan.

### 4.2. Selection of Immobilizing Agents (Biochar and Gravel Sand) and Experimental Setup

Biochar derived from sugarcane bagasse was prepared in a laboratory muffle furnace at the pyrolysis temperature of 300 °C, following Sanchez et al. [84]. Biochar produced at 300 °C effectively immobilizes various heavy metals including Cd [85,86], while that produced at higher temperatures has a reduced proportions of C-containing functional groups which are responsible for metal adsorption [87]. Briefly, after pyrolysis of feedstock, biochar was cooled to room temperature and passed through a 0.25-mm sieve. An analysis of its physicochemical properties is shown in Table 3. Electrical conductivity (EC) and pH were examined by preparing a 1:20 (*w/v*) solution, and cation exchange capacity (CEC) followed the NH_4_-acetate method described by Gaskin et al. [88]. Moisture content was measured following Enders and Lehmann [89] using the following equation;

*Moisture contents* (%) = {(*weight _as received_* − *weight _105 °C dried_*)/*weight _as received_*} × 100 (1)

Gravel sand (2 mm) used in this experiment was purchased from the Pakistan Minerals Company, Karachi, Pakistan.

The soil was artificially spiked with Cd by adding cadmium chloride (CdCl_2_) salt at the rate of 60 mg kg^−1^ and by mixing continuously for two weeks for uniform distribution of the salt. Gravel sand and biochar were added at the rate of 1% (*w/w*). Polythene lined pots were filled with 8 kg of the prepared soil mixes. There were 24 pots representing inoculated v/s uninoculated pots under each applied treatment. Five seeds were sown in each pot, and after germination, the three most vigorous plants were maintained. The following four treatments, each with and without bacterial inoculation, were arranged in a completely randomized design (factorial) with three replications as T1: control; T2: biochar; T3: gravel sand; and T4: biochar and gravel sand. The pots were placed in the rain protected wire-house of ISES, UAF with no control on natural light, humidity, and temperature throughout the growth period.

### 4.3. Biomass and Physiological Measurements

On day 45 after germination, the physiological parameters, net assimilation rate (A*n*), internal carbon dioxide concentration (C*i*), and evapotranspiration rate (ET) were determined using a portable infrared gas analyzer (IRGA model LCA-4, Germany). Soil Plant Analysis Development (SPAD) index was estimated from the top 2nd and 3rd fully expanded youngest leaves of each plant using a portable chlorophyll meter (SPAD-502-meter Minolta, Osaka, Japan). Five readings were taken for each leaf and then averaged to assess the physiological gas exchange attributes and SPAD index.

On day 65 after germination, the plants were harvested. Plant growth was assessed from plant height (cm), dry weight of shoots (total above ground biomass) and roots (g·pot^−1^), and seed weight.

### 4.4. Chemical and Biochemical Analyses of Pea Seeds

Determination of Fe and Zn in the pea seeds was carried out by digesting a known weight of grains in a di-acid mixture having the ratio 2:1 (HNO_3_: HClO_4_) [90]. Biochemical parameters (protein, ash, fiber, and fats) of pea seeds were measured according to the standard protocols. The concentration of protein in seed samples was evaluated following the method of Bradford [91]. Pea seed samples were analyzed for ash, fat, and fiber following the methods of AOAC [92]. 

Ash analysis of grain was done following below-mentioned equation:

(2) $$Ash\;\left(\%\right)=\frac{Weight\;of\;ash\;}{Weight\;of\;sample\;}\times 100$$

The ether extract method was used for fat determination by using a Soxhlet apparatus and by applying the following equation:

(3) $$Fat\;\left(\%\right)=\frac{Weight\;of\;ether\;extract\;}{Weight\;of\;sample\;}\times 100$$

All devices utilized for chemical and biochemical examinations were soaked in diluted HNO_3_ (pro analysis quality, Merck) and washed with deionized water before use.

### 4.5. Persistence of Inoculant Strain MN17 in the Rhizosphere, Root, and Shoot Tissues

The inoculant strain was recovered from the rhizosphere, root, and shoot tissues of pea plants following dilution and plate counting technique. Rhizosphere (5 g) and root/shoot (2 g) samples were obtained and were homogenized by mixing with 15 mL of 0.9% (*w*/*v*) NaCl solution and agitation (180 rpm) for 30 min at 28 °C. After settling suspensions, serial dilutions up to 10^−6^ were plated onto a 10% tryptic soya agar (TSA) medium. The plates were incubated at 28 ± 2 °C for 48 hours and colonies were counted to determine the colonization value (CFU per g dry soil/tissues weight). Twenty colonies from each treatment were randomly selected, and their identity as the inoculant strain was confirmed by restriction fragment length polymorphism (RFLP) analysis of the 16S–23S rRNA intergenic spacer (IGS) region [93].

### 4.6. Cd Concentration in Soil and Plant Tissues

For determination of Cd in soil, 10 g air-dried soil subsamples were placed in 20 mL of DTPA solution (0.05 mol L^−1^ at pH 7.3) and shaken on a reciprocal shaker at 200 rpm for 15 min [94]. Cadmium concentration in soil samples was then measured directly from soil extracts after filtration by AAS. For determination of Cd concentration in plants tissues, samples were dried in an oven at 70 °C for 24 h and then ground, weighed, and digested with 2 mL nitric acid (HNO_3_) and 1 mL per chloric acid (HClO_4_) (2:1 ratio *v*/*v*). Afterward, samples were heated on a hot plate at 350 °C until dense white fumes appeared. The contents of these flasks were cooled, filtered, and stored in plastic bottles for further determinations by AAS (Perkin Elmer Aanalyst-100, Waltham, USA) [95]. 

### 4.7. Statistical Analysis

The data were subjected to variance analysis [96] by using software SPSS version 22. Significant differences between treatment means were calculated by Duncan’s Multiple Range Test (DMRT) at *p* ≤ 0.05. Graphs were drawn using computer-based software (Origin Pro 9.1), and principal component analysis (PCA) was performed using XLSTAT software (version 2014).

## 5. Conclusions 

This study clearly demonstrated that the detrimental effects of Cd in soil on pea plant growth and biochemical and nutritional properties can be ameliorated by applying metal immobilizing amendments such as biochar and gravel sand, either alone or in combination. These positive effects are considerably enhanced by the coapplication of *Enterobacter* sp. MN17 as a seed inoculant. Both the applied biochar and gravel sand efficiently immobilized Cd in soil by reducing its concentration in the soil and its uptake by the plants. We conclude that the combined application of biochar, gravel sand, and *Enterobacter* sp. MN17 inoculation is a valuable, environment-friendly solution to ameliorate soils contaminated with Cd for safer production of crops, especially vegetables. 

## Figures and Tables

**Table 1:** Effects of immobilizing agents (biochar and gravel sand) with and without seed inoculation of bacterial strain MN17 on biochemical and nutritional parameters of pea plants.

Treatments	Protein (%)	Fat (%)	Fiber (%)	Ash (%)	Fe in Seeds (mg kg^−1^)	Zn in Seeds (mg kg^−1^)
Control	11.60 ± 0.81 d †	0.82 ± 0.02 c	3.93 ± 0.26 d	2.20 ± 0.15 d	33.77 ± 1.96 f	15.67 ± 1.31 e
BC	12.80 ± 1.15 cd	0.94 ± 0.01 bc	4.33 ± 0.20 bcd	2.43 ± 0.30 c	40.53 ± 1.42 e	18.30 ± 1.36 bcd
GS	12.40 ± 1.10 cd	0.91 ± 0.02 c	4.10 ± 0.2 cd	2.40 ± 0.2 cd	39.50 ± 0.85 e	17.70 ± 1.17 cd
BC + GS	13.67 ± 1.05 c	1.03 ± 0.05 bc	4.50 ± 0.2 bcd	3.09 ± 0.20 b	49.63 ± 1.30 c	19.71 ± 1.65 bc
MN17	13.43 ± 1.09 cd	1.01 ± 0.08 bc	4.30 ± 0.3 bcd	2.43 ± 0.15 c	42.53 ± 1.1 de	17.50 ± 0.7 cd
BC + MN17	15.50 ± 1.57 ab	1.33 ± 0.02 a	4.86 ± 0.05 ab	3.35 ± 0.25 ab	54.40 ± 1.62 b	21.07 ± 1.51 ab
GS + MN17	14.63 ± 1.20 abc	1.23 ± 0.03 ab	4.68 ± 0.2 abc	2.96 ± 0.15 b	47.80 ± 0.65 cd	19.98 ± 0.83 bc
BC + GS + MN17	16.40 ± 1.03 a	1.43 ± 0.02 a	5.17 ± 0.2 a	3.79 ± 0.26 a	62.23 ± 0.45 a	22.97 ± 1.74 a

† Means sharing the same letters in a column do not differ significantly at *p* < 0.05. The values are mean ± S.D. (*n* = 3). BC: biochar; GS: gravel sand; BC + GS: biochar + gravel sand; MN17: *Enterobacter* sp. MN17.

**Table 2:** Pearson correlation among the plant growth, physiological and biochemical parameters, and Cd concentration in soil and plant tissues.

Variables	PH	SDW	RDW	GW	Pro	Fat	Fib	Ash	G-Fe	G-Zn	Cd-So	Cd-Sh	Cd-R	Cd-G	Ci	An	ET	SPAD
**PH**	**1**																	
**SDW**	**0.987**	**1**																
**RDW**	**0.989**	**0.97**	**1**															
**GW**	0.958	0.973	0.941	**1**														
**Pro**	0.975	0.988	0.968	0.982	**1**													
**Fat**	**0.988**	0.994	**0.983**	**0.977**	**0.989**	**1**												
**Fib**	0.949	0.965	0.924	0.919	**0.939**	**0.953**	**1**											
**Ash**	0.933	0.948	0.92	0.897	0.906	**0.942**	**0.979**	**1**										
**G-Fe**	0.93	0.959	0.926	**0.989**	0.971	**0.966**	0.9	0.896	**1**									
**G-Zn**	0.946	0.959	0.929	0.888	0.914	**0.944**	**0.976**	**0.989**	0.879	**1**								
**Cd-So**	**−0.909**	**−0.941**	**−0.898**	**−0.961**	**−0.927**	**−0.94**	**−0.876**	−0.904	**−0.978**	−0.891	**1**							
**Cd-Sh**	−0.83	−0.841	−0.818	−0.782	−0.766	−0.838	**−0.866**	**−0.946**	−0.796	−0.928	**0.865**	**1**						
**Cd-R**	−0.781	−0.807	−0.77	−0.725	−0.725	−0.788	−0.839	−0.923	−0.75	−0.92	0.831	**0.98**	**1**					
**Cd-G**	−0.812	−0.83	−0.802	−0.739	−0.749	−0.809	−0.857	**−0.932**	−0.755	**−0.939**	0.83	**0.974**	**0.996**	**1**				
**Ci**	0.849	0.87	0.837	0.749	0.809	0.835	0.899	**0.919**	0.749	**0.957**	−0.777	**−0.87**	**−0.915**	**−0.941**	**1**			
**An**	0.966	0.979	0.948	**0.952**	0.971	**0.978**	**0.988**	**0.957**	0.932	**0.952**	**−0.89**	−0.825	−0.78	−0.799	0.849	**1**		
**ET**	0.955	0.955	**0.944**	0.917	**0.935**	**0.951**	**0.989**	**0.977**	**0.9**	**0.968**	**−0.873**	**−0.865**	−0.834	−0.856	0.89	**0.976**	**1**	
**SPAD**	0.818	0.84	0.791	0.781	0.793	0.828	**0.95**	**0.95**	0.771	0.922	−0.759	**−0.872**	−0.854	−0.857	0.863	0.905	**0.939**	**1**

Values in bold shows significant correlation (*p* = 0.05) among corresponding plant growth, physiological and biochemical parameters, and pea tissues Cd contents under Cd-contaminated soil conditions. PH: plant height; SDW: shoot dry weight; RDW: root dry weight; GW: grains weight; Pro: protein; Fat: fat; Fib: fiber; Ash: ash; G-Fe: Fe concentration in grains; G-Zn: Zn concentration in grains; Cd-So: Cd concentration in soil; Cd-Sh: Cd concentration in shoot; Cd-R: Cd concentration in roots; Cd-G: Cd concentration in grains; Ci: internal carbon dioxide concentration; An: net assimilation rate; ET: evapotranspiration rate; and SPAD: chlorophyll (SPAD) contents.

**Table 3:** Physicochemical characteristics and nutritional composition of sugarcane bagasse biochar and soil utilised in this study.

Physical/Chemical Properties	Soil	Biochar
Textural class	Sandy clay loam	-
Sand (%)	50 ± 1.2	-
Silt (%)	35 ± 0.05	-
Clay (%)	15 ± 1.70	-
pH	7.33 ± 0.04	6.50 ± 0.05
Electrical conductivity (dS m^−1^)	2.45 ± 0.01	1.61 ± 0.04
Cation exchange capacity (cmol_c_ kg^−1^)	6.89 ± 1.46	88.40 ± 2.2
Moisture %	30 ±1.33	3.10 ± 0.11
Soluble carbonates (mmol_c_ L^−1^)	0.88 ±0.42	-
Soluble bicarbonates (mmol_c_ L^−1^)	*ND*	-
Organic matter (%)	0.77 ± 0.12	-
Organic carbon (%)	-	60.51 ± 0.70
Nitrogen (%)	0.046 ± 0.47	1.59 ± 0.01
Available phosphorous (mg kg^−1^)	3.40 ± 1.12	-
Total phosphorus (g kg^−1^)	-	3.21 ± 0.42
Extractable potassium (mg kg^−1^)	110 ± 2.10	-
Total potassium (g kg^−1^)	-	2.01 ± 0.18
Zinc (mg kg^−1^)	-	77.32 ± 3.21
Iron (mg kg^−1^)	-	110.31 ± 5.31
Total cadmium (mg kg^−1^)	0.53 ± 0.10	*ND*

The values are mean ± S.E. (*n* = 3). *ND*: not detected; - parameters not measured.
